# U-shaped association between low-density lipid cholesterol and diabetes mellitus in patients with hypertension

**DOI:** 10.1186/s12944-019-1105-5

**Published:** 2019-08-17

**Authors:** Lin Liu, Geng Shen, Jia-yi Huang, Yu-ling Yu, Chao-lei Chen, Yu-qing Huang, Ying-qing Feng

**Affiliations:** 1 The Second School of Clinical Medicine, Southern Medical University, Guangzhou, China; 2 School of Medicine, South China University of Technology, Guangzhou, China; 3 Department of Cardiology, Guangdong Cardiovascular Institute, Hypertension Research Laboratory, Guangdong Provincial People’s Hospital, Guangdong Provincial Key Laboratory of Coronary Heart Disease Prevention Guangdong Academy of Medical Sciences, South China University of Technology School of Medicine, The Second School of Clinical Medicine, Southern Medical University, Guangzhou, 510080 China

**Keywords:** Low-density lipid cholesterol, Diabetes mellitus, Hypertension

## Abstract

**Background:**

The magnitude and direction of association of low-density lipid cholesterol (LDL-C) with diabetes mellitus (DM) might differ by hypertensive status, but there is limited epidemiological evidence in China.

**Methods:**

We examined the association between LDL-C levels and DM in 9892 participants with hypertension using logistic regression. Participants were stratified into three groups according to LDL-C levels (desirable, borderline high or high), then further divided into quartiles. Restricted cubic spline regression models, subgroup analysis and interaction tests were also conducted to evaluate the shape of association.

**Results:**

After adjusting for covariates, lower LDL-C had a significant and inverse association with the likelihood of DM in all participants (OR: 0.944, 95% CI = 0.893, 0.998). In participants with desirable LDL-C concentrations (< 3.4 mmol/L), LDL-C protected against DM (OR = 1.240, 95% CI = 1.076, 1.429 per 1 mmol/L decrease). In participants with higher LDL-C concentrations (> 4.1 mmol/L), LDL-C increased the DM likelihood (OR = 1.536, 95% CI = 1.126, 2.096 per 1 mmol/L increase). Restricted cubic spline regression also found a U-shaped association between LDL-C levels and DM prevalence.

**Conclusions:**

There was a U-shaped association between LDL-C levels and DM in Chinese patients with hypertension.

**Electronic supplementary material:**

The online version of this article (10.1186/s12944-019-1105-5) contains supplementary material, which is available to authorized users.

## Background

The number of adults with diabetes mellitus (DM) worldwide was estimated to be 366 million in 2011 and is expected to reach 552 million by 2030 [1, 2]. The International Diabetes Federation has estimated that 50% of adults with diabetes are undiagnosed [3]. DM is also highly prevalent in China and is a great disease burden [4]. Lowering low-density lipoprotein cholesterol (LDL-C) has been considered to be essential in preventing cardiovascular disease [5]. However, statin therapy has been associated with a slightly increased risk of DM despite unknown clinical significance. [6, 7] The relationship between statins and DM is also subject to “reverse epidemiology” or the “risk factor paradox” observed in obese or geriatric populations [8, 9]. In China, low LDL-C levels appeared to associate with an increased risk of intracerebral hemorrhage [10], while high triglycerides (TG) associated with a decreased risk of cognitive decline and daily activity, as well as an increased risk of frailty and mortality in elderly [11]. Meanwhile, data from representative cross-sectional studies in the USA and Spain indicated an positive association between LDL-C levels and body mass index (BMI) only in lean individuals [12]. Moreover, lipid paradox have been observed in hypertensive populations [13–15]. In addition to obesity, hypertension may also affect cholesterol metabolism, but the impact of LDL-C on DM occurrence has received less attention, particularly among patients with hypertension.

## Methods

### Study design and participants

We enrolled 10,322 participants over 18 years of age who were included in a registry of hypertensive patients in Dongguan, Guangdong, China in 2015. A total of 9892 patients with a complete set of data were included in the analysis. The present study complied with the ethical principles of the Declaration of Helsinki and was approved by the institutional medical ethical committee at Guangdong General Hospital, Guangzhou, China. All enrolled participants have provided written informed consent.

### Collection of participant data

A structured questionnaire was administered by trained staff to obtain participants’ age, sex, smoking status, alcohol consumption, medical history including coronary artery disease (CAD), DM, and stroke, and medication history including the use of β-blockers, calcium channel blockers (CCB), angiotensin-converting enzyme inhibitors (ACEI) / angiotensin-receptor blockers (ARB), and statins. Participants’ height, weight, waist circumference (WC), systolic blood pressure (SBP), diastolic blood pressure (DBP), fasting blood glucose (FBG), total cholesterol (TC), triglycerides (TG), low-density lipoprotein cholesterol (LDL-C), high-density lipoprotein cholesterol (HDL-C), estimated glomerular filtration rate (eGFR), and BMI (kg/m^2^) were assessed. eGFR was calculated as 186 × Scr^− 1.154^ × age^− 0.203^ × 0.724 for women; Scr is serum creatinine (mg/dL). Following the 2010 Chinese guidelines for the management of hypertension [16], hypertension was defined as SBP ≥ 140 mmHg, DBP ≥ 90 mmHg, and/or use of antihypertensive medications within 2 weeks of enrollment. Participants were considered to have DM if they had previously been diagnosed by a registered medical practitioner, and/or had used hypoglycemic drugs within 2 weeks, and/or had a baseline FBG ≥ 7.0 mmol/l. Dyslipidemia was defined as TC ≥ 6.2 mmol/L (high TC), and/or TG ≥ 2.3 mmol/L (high TG), and/or LDL-C (high LDL-C) ≥ 4.1 mmol/L, and/or HDL-C < 1.0 mmol/L (low HDL-C), and/or use of lipid-lowering medications [17].

### Statistical analysis

Categorical variables were reported as numbers and percentages and compared by the *χ*^*2*^ test. Continuous values were reported as means ± standard deviation and compared using the Kruskal–Wallis test or one-way analysis of variance. Logistic regression was used to estimate the association between LDL-C level and DM in all participants. The study participants were stratified into three groups by their LDL-C levels, namely desirable (< 3.4 mmol/L), borderline high (3.4 to 4.1 mmol/L) and high (> 4.1 mol/L) as defined according to the 2016 Chinese guidelines for the management of dyslipidemia in adults [17]. Logistic regression was used to estimate the odds ratios (ORs) and 95% confidence Intervals (CIs) of DM in each group. Logistic regression was also used to calculate the *p-values* for trends of the quartiles in each LDL-C concentration. We have built 3 regression models for each stratum of LDL-C concentration. Model 1 only included LDL-C concentration, model 2 was additionally adjusted for age and sex, and model 3 was additionally adjusted for smoking status, alcohol consumption, history of CAD and stroke, use of *β*-blockers, CCB, ACEI/ARB, statins, BMI, WC, SBP, DBP, TG, HDL-C and eGFR. The shape of association between LDL-C levels and DM was examined by restricted cubic spline regression models. The interactions of LDL-C level with age, sex, smoking, alcohol consumption, history of CAD or stroke, statin usage and BMI were evaluated by interaction tests. Multiple imputation based on five replications and a chained equation method using the R statistics “mice” package was used to account for missing data with 1234 as a random number seed [18]. *P* values less than 0.05 were considered statistically significant. R 3.5.1 (https://cran.r-project.org/mirrors.html) was used for all statistical analysis.

## Results

### Participant characteristics

Table 1 shows the demographic characteristics of the 9892 participants stratified by their LDL-C levels. The participants included 4921 men, and were 62.9 ± 13.7 years of age, 23.7% of them had DM and 58.7% had dyslipidemia. Participants with LDL-C concentrations ≥4.1 mmol/L were on average older in age, higher proportion being women, and had higher WC, SBP, TC, TG, HDL-C, and FBG values, higher prevalence of DM and CAD, and lower baseline eGFR than participants with LDL-C concentrations 3.4–4.1 mmol/L. Participants with LDL-C concentrations < 3.4 mmol/L were younger in age, had higher FBG, lower WC and SBP than participants in other two groups, and higher prevalence of DM or CAD.

**Table 1:** Participants characteristics

Characteristics	Overall	LDL-C, mmol/L			
		< 3.4	3.4 to 4.1	≥4.1	*P*-value
No. of participants	9892	5038	2847	2007	
Demographics					
Age, y	62.9 ± 13.7	61.9 ± 14.3	62.9 ± 13.4	65.2 ± 12.4	< 0.001
Male	4921 (49.7%)	2651 (52.6%)	1420 (49.9%)	850 (42.4%)	< 0.001
Smoking	2622 (26.5%)	1328 (26.4%)	773 (27.2%)	521 (26.0%)	0.615
Alcohol use	1388 (14.0%)	686 (13.6%)	452 (15.9%)	250 (12.5%)	0.002
BMI, kg/m2	24.8 ± 3.85	24.8 ± 3.89	24.8 ± 3.75	25.0 ± 3.90	0.028
Waist circumference	88.0 ± 9.63	87.8 ± 9.79	88.0 ± 9.40	88.6 ± 9.52	0.016
Comorbidity					
Stroke	205 (2.1%)	134 (2.7%)	45 (1.6%)	26 (1.3%)	< 0.001
Coronary artery disease	161 (1.6%)	103 (2.0%)	26 (0.9%)	32 (1.6%)	< 0.001
Diabetes mellitus	2341 (23.7%)	1298 (25.8%)	599 (21.0%)	444 (22.1%)	< 0.001
Dyslipidemia	5811 (58.7%)	2604 (51.7%)	1200 (42.1%)	2007 (100%)	< 0.001
TC ≥ 6.2 mmol/L	1995 (20.2%)	85 (1.7%)	344 (12.1%)	1566 (78.0%)	< 0.001
TG ≥ 2.3 mmol/L	2165 (21.9%)	1047 (20.8%)	647 (22.7%)	471 (23.5%)	0.021
HDL-C < 1.0 mmol/L	1288 (13.0%)	888 (17.6%)	291 (10.2%)	109 (5.4%)	< 0.001
Medication					
β-blockers	721 (7.3%)	439 (8.7%)	168 (5.9%)	114 (5.7%)	< 0.001
Calcium channel blockers	3139 (31.7%)	1730 (34.3%)	882 (31.0%)	527 (26.3%)	< 0.001
ACEI/ARB	4315 (43.6%)	2417 (48.0%)	1156 (40.6%)	742 (37.0%)	< 0.001
Statin	1898 (19.2%)	1415 (28.1%)	316 (11.1%)	167 (8.3%)	< 0.001
Blood pressure, mm Hg					
Systolic	132 ± 16.2	131 ± 15.8	132 ± 16.3	133 ± 17.0	< 0.001
Diastolic	80.8 ± 15.1	80.5 ± 14.1	81.4 ± 18.9	80.7 ± 11.1	0.04
Biomarkers					
TC,mmol/L	5.31 ± 1.16	4.51 ± 0.742	5.66 ± 0.558	6.83 ± 0.866	< 0.001
TG,mmol/L	1.83 ± 1.58	1.82 ± 1.73	1.83 ± 1.37	1.88 ± 1.46	< 0.001
LDL-C,mmol/L	3.40 ± 0.927	2.69 ± 0.483	3.72 ± 0.221	4.75 ± 0.586	< 0.001
HDL-C,mmol/L	1.35 ± 0.365	1.32 ± 0.365	1.35 ± 0.318	1.41 ± 0.419	< 0.001
FBG,mmol/L	5.35 ± 1.81	5.35 ± 1.79	5.27 ± 1.68	5.47 ± 2.05	0.007
eGFR,ml/min/1.73m^2^	88.4 ± 26.0	89.4 ± 27.4	88.3 ± 24.8	86.0 ± 24.0	< 0.001

BMI indicates body mass index; ACEI, angiotensin-converting enzyme inhibitors; ARB, angiotensin-receptor blockers; TC, total cholesterol; TG, triglyceride; LDL-C, low density lipoprotein-cholesterol; HDL-C, high density lipoprotein-cholesterol; FBG, fasting blood glucose; eGFR, estimated glomerular filtration rate

Values for categorical and continuous variables are given as numbers (percentages) and as means ± standard deviation, respectively

### Association between LDL-C and DM

**Fig. 1:**
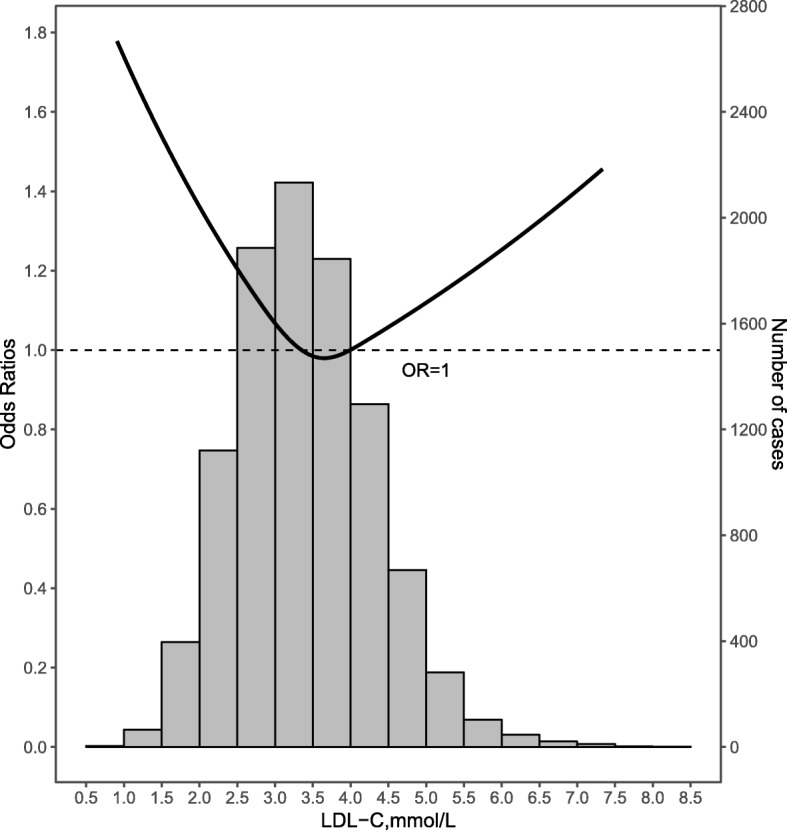
Restricted cubic spline of low-density lipid cholesterol levels and diabetes mellitus. Adjusted for age, sex, smoking, alcohol consumption, history of CAD and stroke, consumption of β-blockers, CCB, ACEI/ARB, statin, BMI, WC, SBP, DBP, TG, HDL-C and eGFR. The solid curve shows the association between LDL-C levels and DM. The histograms below show the distributions of LDL-C level in the populations

Logistic regression found an inverse association between LDL-C and DM but the association was U-shaped after stratification when including LDL-C as a continuous or categorical variable. For participants with LDL-C concentrations < 3.4 mmol/L, there was an inverse association between the lowest quartile of LDL-C and DM likelihood in all regression models when using the highest quartile as referent. For participants with borderline high LDL-C, the highest quartile of LDL-C associated with a lower DM prevalence comparing with the lowest quartile. In participants with high LDL-C concentrations, the highest quartile of LDL-C associated with a higher DM likelihood comparing with the lowest quartile (Tables 2 and 3). The *p*-value for trend was significant in all regression models except for model 3 of participants with borderline high LDL-C. The results of a restricted cubic spline regression shown in Fig. 1 revealed a U-shaped relationship of DM prevalence with increasing LDL-C after adjusting for all covariates.

**Table 2:** Logistic OR (95% CIs) for diabetes mellitus and continuous measures of LDL-C

	No. of participants	model1	model2	model3
Overall (per 1 mmol/L increase)	9892	0.88(0.837,0.926)**	0.893(0.848,0.94)**	0.944(0.893,0.998)**
Per 1 mmol/L decrease in first group (LDL-C < 3.4 mmol/L)	5038	1.364(1.201,1.550)*	1.380(1.213,1.569)*	1.240(1.076,1.429)**
Per 1 mmol/L increase in second group(3.4 ≤ LDL-C < 4.1 mmol/L)	2847	0.657(0.436,0.990)*	0.700(0.463,1.057)*	0.746(0.485,1.149)
Per 1 mmol/L increase in third group (LDL-C ≥ 4.1 mmol/L)	2007	1.270(1.073,1.503)**	1.489(1.108,2.003)**	1.536(1.126,2.096)**

model1, unadjusted;

model2, adjusted for age, sex;

model3, adjusted for age, sex,smoking, alcohol consumption, history of CAD, stroke, consumption of β-blockers, calcium channel blockers, ACEI/ARB, statin, BMI, SBP, DBP, TG, HDL-C and eGFR;

*p < 0.05, **p < 0.01;OR, odds ratio; CIs, confidence Intervals

**Table 3:** Logistic OR (95% CIs) for diabetes mellitus and LDL-C stratified by quartile

	case/total	model1		model2		model3	
		OR(95%CIs)	*p*-value	OR(95%CIs)	*p*-value	OR(95%CIs)	*p*-value
LDL-C < 3.4 mmol/L,*n* = 5038							
Q1	337/1260	1.425(1.192,1.702)	< 0.001	1.441(1.205,1.723)	< 0.001	1.248(1.026,1.517)	0.027
Q2	332/1259	1.195(0.997,1.433)	0.054	1.194(0.996,1.432)	0.056	1.146(0.944,1.391)	0.168
Q3	298/1257	1.037(0.862,1.247)	0.701	1.026(0.853,1.235)	0.784	1.031(0.848,1.253)	0.761
Q4	291/1262	ref		ref		ref	
P for trend		< 0.001		< 0.001		0.015	
3.4 mmol/L ≤ LDL-C < 4.1 mmol/L,*n* = 2847							
Q1	171/712	ref		ref		ref	
Q2	142/712	0.795(0.618,1.022)	0.074	0.814(0.632,1.049)	0.112	0.806(0.618,1.051)	0.111
Q3	154/712	0.880(0.687,1.128)	0.313	0.899(0.700,1.153)	0.401	0.936(0.721,1.215)	0.619
Q4	131/711	0.708(0.548,0.915)	0.008	0.735(0.568,0.951)	0.019	0.753(0.575,0.986)	0.039
P for trend		0.023		0.047		0.105	
LDLC ≥4.1 mmol/L,*n* = 2007							
Q1	103/502	ref		ref		ref	
Q2	103/502	1.012(0.746,1.374)	0.938	1.043(0.766,1.42)	0.789	0.999(0.721,1.383)	0.994
Q3	102/501	0.990(0.729,1.346)	0.950	1.005(0.737,1.37)	0.975	0.990(0.715,1.371)	0.951
Q4	135/502	1.425(1.063,1.910)	0.018	1.489(1.108,2.003)	0.008	1.536(1.126,2.096)	0.007
P for trend		0.024		0.013		0.008	

model1, unadjusted;

model2, adjusted for age, sex;

model3, adjusted for age, sex, smoking, alcohol consumption, history of CAD, stroke, consumption of β -blockers, calcium channel blockers, ACEI/ARB, statin, BMI, SBP, DBP, TG, HDL-C and eGFR;

*OR,* odds ratio, *CIs,* confidence Intervals

### Subgroup analysis

The associations between LDL-C and DM likelihood were stratified by participants’ age, sex, smoking status, alcohol consumption, history of CAD or stroke, BMI, and statin use (Table 4). In patients with desirable LDL-C levels, the inverse association between LDL-C and DM was significantly modified by age and history of stroke (*p* for interaction both < 0.05). In participants with high LDL-C levels, the positive association between LDL-C and DM was modified by the use of statins (*p* for interaction = 0.049). Table 2 and Additional file 1: Table S1 shows that imputation of missing covariate data did not lead to any substantial changes in the results of the analysis.

**Table 4:** Association between LDL-C levels and diabetes mellitus among subgroups

subgroup	LDL-C < 3.4 mmol/L			3.4 mmol/L ≤ LDL-C < 4.1 mmol/L			LDLC ≥4.1 mmol/L		
	OR(95%CIs)	*P*-value	P-interaction	OR(95%CIs)	*P*-value	P-interaction	OR(95%CIs)	*P*-value	P-interaction
Age,y			0.022			0.119			0.648
≥60	0.778(0.642,0.944)	0.011		0.603(0.332,1.094)	0.096		1.316(1.045,1.656)	0.019	
< 60	0.827(0.657,1.040)	0.104		0.885(0.440,1.779)	0.731		1.45(1.044,2.012)	0.026	
Sex			0.771			0.358			0.197
Male	0.774(0.633,0.946)	0.012		0.913(0.48,1.735)	0.78		1.528(1.153,2.026)	0.003	
Female	0.862(0.703,1.057)	0.152		0.616(0.34,1.115)	0.11		1.202(0.949,1.524)	0.127	
Smoke			0.695			0.196			0.265
Yes	0.658(0.490,0.885)	0.006		1.131(0.489,2.615)	0.773		1.580(1.096,2.277)	0.014	
No	0.840(0.713,0.989)	0.036		0.619(0.372,1.029)	0.064		1.240(1.008,1.525)	0.041	
Alcohol consumption			0.333			0.395			0.399
Yes	0.645(0.428,0.972)	0.036		1.166(0.344,3.952)	0.805		1.489(0.849,2.612)	0.165	
No	0.831(0.713,0.967)	0.017		0.702(0.440,1.12)	0.137		1.285(1.063,1.555)	0.01	
CAD			0.293			0.114			0.639
Yes	0.967(0.265,3.531)	0.959		0.186(0,Inf)	1		0.195(0,Inf)	1	
No	0.802(0.695,0.926)	0.003		0.777(0.504,1.199)	0.255		1.32(1.102,1.581)	0.003	
Stroke			0.005			0.219			0.126
Yes	3.547(1.236,10.181)	0.019		0.000(0.000,50.287)	0.123		0(0,Inf)	1	
No	0.775(0.671,0.895)	0.001		0.763(0.494,1.179)	0.223		1.288(1.076,1.542)	0.006	
BMI, kg/m2			0.411			0.099			0.417
≥24	0.827(0.691,0.990)	0.039		0.764(0.448,1.302)	0.322		1.349(1.083,1.681)	0.008	
< 24	0.993(0.914,1.079)	0.869		0.597(0.273,1.306)	0.197		1.314(0.952,1.815)	0.097	
Statin			0.622			0.748			0.049
Yes	0.996(0.990,1.002)	0.207		1.000(0.969,1.031)	0.982		1.027(1.009,1.045)	0.003	
No	0.994(0.989,0.999)	0.010		0.992(0.981,1.004)	0.216		1.005(1.000,1.010)	0.034	

LDL-C,low density lipoprotein-cholesterol, CAD, coronary artery disease, BMI, body mass index, OR, odds ratio; CIs, confidence Intervals

model adjusted for age, sex, smoking, alcohol consumption, history of CAD, stroke, consumption of β-blockers, calcium channel blockers, angiotensin-converting enzyme /angiotensin-receptor blockers, statin, BMI, systolic blood pressure, diastolic blood pressure, TG, HDL-C and estimated glomerular filtration rate

## Discussion

The present study revealed a U-shaped association between LDL-C levels and the chance of DM among hypertensive participants in China. Among participants with desirable LDL-C concentration and aged over 60, LDL-C was protective against DM. Among participants with high LDL-C concentration, LDL-C increased the likelihood of DM even with the use of statin. The applicability of reducing LDL-C as much as possible may vary by age, stroke history and the use of statin.

The inverse association between LDL-C level and DM among participants with desirable LDL-C concentration may be related to statin therapy. Statin therapy was associated with a 9% increased risk of incident DM, with the effect being stronger in older individuals [6]. Inhibition of 3-hydroxy-3-methylglutaryl-CoA reductase (HMGCR) increases the expression of LDL receptors in many tissues and promotes transmembrane cholesterol transport. This alteration in cholesterol transport may be involved in the pathogenesis of diabetes [19]. Nevertheless, after stratifying by the use of statin, multivariable logistic regression found that the relationship between DM and LDL-C was not affected by statins. Genetic studies have shown that the incidence of new-onset DM is associated with low LDL-C levels [19, 20], which are consistent with a previous finding that patients with familial hypercholesterolemia were less likely to have type 2 diabetes [21]. In addition, overexpression of Niemann-Pick C1-like 1 (*NPC1L1*), an inhibitor of the LDL-C transporter, may suppress gluconeogenesis. In contrast, inhibiting *NPC1L1* by ezetimibe, a lipid-lowering agent, may increase gluconeogenesis [22].

Overweight and obesity may have influenced the associations between LDL-C and the likelihood of DM. The average BMI of our participants with desirable LDL-C concentration was approximately 25 kg/m^2^. Insulin resistance is physiologically linked with body weight gain, while statins was also associated with increased BMI and body weight gain hence the incidence of DM in a previous study [23]. It was also reported that LDL-C decreased among participants with BMIs > 27 kg/m^2^ [12], indicating that the reverse relationship between decreased LDL-C levels and DM in participants with desirable LDL-C levels may have been associated with high BMIs. Adipocytes are passive energy storage tissues that produce adipocytokines that protect against the establishment of insulin resistance in the liver and skeletal muscle. Obesity or insulin resistance may result in decreased lipid deposition leading to consistent reduction in LDL-C particle formation [24].

Moreover, age may have affected the relationship between LDL-C levels and the chance of getting DM. Changes in TC, TG and LDL-C levels that occur in the geriatric population have been inversely associated with all-cause mortality. The association might be a result of decreased cellular antioxidant capacity and LDL-C production described in the free-radical theory of aging (FRTA) [25–28]. However, the relationship between LDL-C levels and DM in geriatric populations has not been reported. We found a inverse association between LDL-C and DM in older, hypertensive participants with low LDL-C levels, but it is not clear whether FRTA can account for this relationship.

Hypertensive patients with LDL-C levels ≥4.1 mmol/L had an increased chance of having DM. A longitudinal study in Iran found that a high LDL-C level was significantly associated with increased risk of new-onset DM, which is consistent with our results [29]. In this study, the impact of higher LDL-C levels on DM likelihood was stronger in participants who were using statins than in those who were not. The result indicates that the increase of prevalent DM in participants using statins may not have been associated with decreased LDL-C levels. The association between LDL-C levels ≥4.1 mmol/L and an increased chance of DM may result from an interaction of health status with changes in metabolism. The physiology of that relationship needs further investigation.

The main strength of our study is that we fitted restricted cubic spline models to demonstrate a U-shaped association between LDL-C levels and DM. However, the limitation of this study was the use of cross-sectional data. Further longitudinal studies are needed to verify the associations that we found. Secondly, there might have residual confounding effects because DM is a multifactorial, heterogeneous disease. Thirdly, findings in this population is not generalizable to people with different ethnic backgrounds and disease status. Fourthly, we did not assess different types of DM and how it might relate to cholesterol metabolism.

## Conclusion

This cross-sectional study found a U-shaped relationship between LDL-C levels and the likelihood of DM in a Chinese registry of hypertensive patients. LDL-C was protective against DM in patients with concentrations of < 3.4 mmol/L, but increased the chance of having DM in those with high LDL-C concentrations.

## Additional file

Additional file 1: **Table S1.**Logistic OR (95% CIs) for diabetes mellitus and continuous measures of LDL-C with multiple imputation data, *n* = 10,322 (XLSX 9 kb)

## Data Availability

The datasets used and/or analyzed during the current study are available from the corresponding author on reasonable request.

## Abbreviations

ACEI: Angiotensin-converting enzyme inhibitors
ARB: Angiotensin-receptor blockers
BMI: Body mass index
CAD: Coronary artery disease
CCB: Calcium channel blockers
CIs: Confidence intervals
DBP: Diastolic blood pressure
DM: Diabetes mellitus
eGFR: estimated glomerular filtration rate
FBG: Fasting blood glucose
FRTA: Free radical theory of aging
HDL-C: High-density lipoprotein cholesterol
HMGCR: 3-hydroxy-3-methylglutaryl-CoA reductase
LDL-C: Low-density lipoprotein cholesterol
NPC1L1: Niemann-Pick C1-like 1
OR: Odds ratio
SBP: Systolic blood pressure
TC: Total cholesterol
TG: Triglyceride
WC: Waist circumference
